# Comparative Study of Gold and Carbon Nanoparticles in Nucleic Acid Lateral Flow Assay

**DOI:** 10.3390/nano11030741

**Published:** 2021-03-15

**Authors:** Juan Carlos Porras, Mireia Bernuz, Jennifer Marfa, Arnau Pallares-Rusiñol, Mercè Martí, María Isabel Pividori

**Affiliations:** 1 Grup de Sensors i Biosensors, Departament de Química, Universitat Autònoma de Barcelona, 08193 Bellaterra, Spain; juancarlos.porras@e-campus.uab.cat (J.C.P.); Mireia.Bernuz@uab.cat (M.B.); Jennifer.Marfa@uab.cat (J.M.); Arnau.Pallares@uab.cat (A.P.-R.); 2 Institute of Biotechnology and Biomedicine, Universitat Autònoma de Barcelona, 08193 Bellaterra, Spain; merce.marti@uab.cat

**Keywords:** carbon black, gold nanoparticles, rapid diagnostic tests, lateral flow assay

## Abstract

A lateral flow assay (LFA) is a paper-based, point-of-need test designed to detect a specific analyte in complex samples in low-resource settings. Although LFA has been successfully used in different applications, its use is still limited when high sensitivity is required, especially in the diagnosis of an early-stage condition. The limit of detection (LOD) is clearly related to the signal-generating system used to achieve the visual readout, in many cases involving nanoparticles coupled to a biomolecule, which, when combined, provides sensitivity and specificity, respectively. While colloidal gold is currently the most-used label, other detection systems are being developed. Carbon nanoparticles (CNPs) demonstrate outstanding features to improve the sensitivity of this technology by producing an increased contrast in the paper background. Based on the necessity of sensitivity improvement, the aim of this work is a comparative study, in terms of analytical performance, between commercial streptavidin gold nanoparticles (streptAv-AuNPs) and avidin carbon nanoparticles (Av-CNPs) in a nucleic acid lateral flow assay. The visual LOD of the method was calculated by serial dilution of the DNA template, ranging from 0.0 to 7 pg μL^−1^/1.5 × 10^4^ CFU mL^−1^). The LFA achieved visual detection of as low as 2.2 × 10^−2^ pg μL^−1^ using Av-CNPs and 8.4 × 10^−2^ pg μL^−1^ using streptAv-AuNPs. These LODs could be obtained without the assistance of any instrumentation. The results demonstrate that CNPs showed an increased sensitivity, achieving the nanomolar range even by visual inspection. Furthermore, CNPs are the cheapest labels, and the suspensions are very stable and easy to modify.

## 1. Introduction

The recent outbreak of SARS-CoV-2 that spread worldwide in a matter of days due to the globalization process highlighted the need for rapid diagnostic tests (RDTs) able to provide a rapid result and to help implement security and prevention measures to interrupt the transmission chain. At present, and more than ever, these RDTs are a major research field of growing interest among researchers around the globe. In 2003, the World Health Organization (WHO/TDR) published a set of criteria for the ideal RDT test under the acronym ASSURED [1]: Affordable, Sensitive, Specific, User-friendly, Rapid and Robust, Equipment-free, and Deliverable to end-users. Although the original features remain relevant, it has been recently revised to include two additional criteria as REASSURED of R (Real-time connectivity) and E (Ease of specimen collection and environmental friendliness), in order to design future devices [2].

Since the presentation of the first US patents by three individual researchers in 1987 (Robert Rosenstein for Becton Dickinson & Co., Keith May for Unilever, and David Charlton for Carter Wallace), lateral flow assays (LFAs) are the most common commercially available RDTs. There has since been an increasing interest in the development of LFAs for multiple chemical and biochemical targets [3]. The LFA in its most general format is an immunochromatographic assay that separates analytes that flow across porous membranes and interact specifically with antibodies deposited on them. It requires a visible signal-generating system to label the analyte, or its receptor, and create a visual result that will be interpreted by the end-user. The most popular types of signal-generating systems based on nanoparticles (NPs) are metallic NPs, such as gold NPs, and polymeric NPs, such as latex or polystyrene [4]. Although LFA is one of the RDT formats that can meet the WHO criteria, its poor sensitivity in some applications [5] is a major drawback which can limit its large-scale use for the global diagnosis of major diseases. The study of alternative NPs as signal-generating systems emerges as a response to this bottleneck. Different approaches have been reported in order to address this issue, including the use of alternative nanoparticles as signal-generating systems, such as quantum dots [6], as well as other labels based on fluorescence [7], chemiluminescence readout [8,9], enzyme-like activity [10], or other readout systems [11,12]. Although these systems provide enhanced sensitivity, in most cases the addition of further reagents or substrates, or the need for instrumentation to achieve the readout, can be a barrier in some low-resource settings.

The term “carbon nanoparticles” (CNPs) generically refers to several types of nanostructured carbon products based on the product carbon black (CB), mainly used in industrial manufacturing processes, such as for rubbers and tires, plastics, inks, paints, and electrodes [13,14]. These extremely fine black powders are 97–99% elemental carbon aggregates ranging from tens to a few hundred nanometers. The microstructure of CNPs has been studied for many years [15,16,17,18], and it is considered to be paracrystalline and highly complex, offering a material with high surface area, high stability, low density, and high mechanical strength [13]. The carbon black aggregates are the primary units that are discrete, rigid colloidal particles formed by fused spheres of graphitic carbon layers [13]. The X-ray diffraction (XRD) patterns of CB define a 2D arrangement of the carbon hexagon layers that are superposed in random orientation with its adjacent layers. Although it cannot be considered an amorphous material, CB does not have a three-dimensional network structure like graphite [16,17,18]. CB is produced by the pyrolysis of hydrocarbons at temperatures above 1200 °C, and different classifications have been proposed according to its production process, its carbon feedstock, its application field, and the properties of the end-of-use products [13].

Carbon nanoparticles and other carbon allotropes are well-known materials in the electrochemical biosensors field for transducers and are used in several applications, from the fabrication of electrodes, such as graphite/epoxy composite (GEC) [19] and screen-printed electrodes [14], to their use as labeling in dot blot and LFAs [20,21], taking advantage of the surface reactivity and high adsorption of carbon [22,23,24,25,26].

Their characteristics and mass-scale production for industrial applications provide a low-cost material with the appropriate reactivity and stability to be used for labeling in LFAs [20,21,22,23,24,25,26]. The intrinsic black color of the CNPs provides the highest possible visual contrast in a white nitrocellulose membrane, therefore enhancing the analytical parameters of LFA [27,28].

This work addresses the development of biologically modified carbon NPs, their characterization by transmission electron microscopy (TEM), and their application in lateral flow assays using *Escherichia coli* double-tagged DNA amplicons [29,30]. Biotin and digoxigenin were used as specific tags for labeling the PCR-amplified DNA. Thus, avidin was used to modify the CNPs’ surface and anti-digoxigenin antibody was deposited on the LFA nitrocellulose membrane. The analytical parameters and performance of biologically modified CNPs are compared with commercially available streptavidin-modified gold NPs as the standard signal-generating system in LFA.

## 2. Materials and Methods

### 2.1. Reagents and Equipment

The carbon nanoparticles (CNPs) obtained from carbon black (CB) Spezial Schwartz 4 (Degussa AG, Essen, Germany, Ref.7088) and avidin (Thermo Scientific, Waltham, MA, USA, Ref. 21121) were used to prepare the avidin-modified carbon nanoparticles (Av-CNPs). Commercial streptavidin-modified 40 nm gold nanoparticles were obtained from Expedeon (Cambridge, UK, Ref. 250–1000). Bovine serum albumin (BSA, Ref. A5403), sucrose (Ref. S0389), and Tween^®^ 20 (Ref. P9416) were purchased from Sigma-Aldrich (St. Louis, MO, USA). All solutions were prepared with ultrapure Milli-Q water (Millipore^®^ System, Burlington, MA, USA, resistivity 18.2 MΩ·cm). The composition of these solutions is described in the Supplementary Materials. For the double-tagging PCR, the tagged primers were obtained from Sigma-Aldrich. Taq DNA polymerase (Ref. 18038067) and nuclease-free water (Ref. AM9937) were purchased from Thermo Fisher, Waltham, MA, USA. Standard reaction buffer 10× with MgCl_2_ (Ref. 20.034-4182) was purchased from Biotools, Madrid, Spain. Deoxynucleotide Mix 10 mM (Ref. D7295) was purchased from Sigma-Aldrich, St. Louis, MO, USA.

For the construction of lateral flow assay (LFA) strips, adhesive backing cards were purchased from Kenosha C.V., Amstelveen, The Netherlands (Ref. KN-2211). Nitrocellulose membrane (Ref. FF120H) and absorbent pads (CF7) were purchased from GE Healthcare Europe, Chicago, IL, USA. The cellulose sample pads (CFSP203000) and glass fiber conjugate pads (GFCP083000) were purchased from Millipore, Burlington, MA, USA. Anti-digoxigenin Fab fragments (anti-DIG antibody) (Roche, Basilea, Switzerland, Ref. 11214667001) were used in the test line at 0.4 mg·mL^−1^. As a control line, biotinylated protein was used at 0.4 mg·mL^−1^ (a biotin-modified rabbit polyclonal antibody) (Abcam, Cambridge, UK, Ref. ab69255).

For the preparation of carbon black conjugates, an Eppendorf Thermomixer C (Eppendorf AG, DE, Hamburg, Germany), a microtube centrifuge Biocen 22R (Ortoalresa, ES), and a probe sonicator (Sonics Materials, Newtown, CT, USA Ref. VCX130PB220) were used. A SimpliAmp Thermal Cycler (Applied Biosystems, Foster City, CA, USA) was used for the double-tagging PCR amplification. For the immobilization of biomolecules into LFA membranes, an IsoFlow reagent dispensing system (Claremont Bio, Upland, CA, USA) coupled with a syringe bomb KDS LegatoTM 200 (KD Scientific Inc., Holliston, MA, USA) were used at a flow rate of 38 μL min^−1^.

### 2.2. Carbon Nanoparticles Modification and Storage

Briefly, 10 mg·mL^−1^ CNP solution was homogenized in Milli-Q water by sonication at 27 W for 5 min and 5-fold diluted in 5 mmol·L^−1^ borate buffer, pH 8.8. The immobilization was performed by physical adsorption [20]. To achieve this, 350 µL of avidin (1 mg·mL^−1^) was added to 1 mL CNP solution and incubated for 3 h at RT using a thermomixer at 550 rpm (slight rotation). The suspension containing the avidin-modified carbon nanoparticles (Av-CNPs) was centrifuged for 15 min at 13,600× *g,* and the supernatant was removed to eliminate the excess avidin. Afterward, Av-CNPs were resuspended in 1 mL of 5 mmol·L^−1^ borate buffer, pH 8.8 with 1% (*w*/*v*) BSA, and centrifuged again under the same conditions. Finally, Av-CNPs were resuspended in 1 mL storage buffer (100 mmol·L^−1^ borate, pH 8.8 with 1% (*w*/*v*) BSA) to a final concentration of 0.2% (*w*/*v*) of Av-CNPs. The final conjugate solution was stored at 4 °C until further use.

### 2.3. Characterization of CNPs by Transmission Electron Microscopy

A JEOL 1400 transmission electron microscope (TEM) at an accelerating voltage of 120 kV was used for the characterization of the CNPs. For sample preparation, a micro-quantity of CNP solution was sonicated at 27 W for 5 min and dropped on a copper plate with a carbon film grid until dried. Images were further processed by the Nucleus counting plugin for ImageJ to calculate their size distribution. Before the analysis, images were transformed to 8-bit, with the contrast and brightness adjusted and binarized to make them processable by the software. Moreover, before the plugin was employed, a watershed filter was applied to separate them correctly. Only well-separated CNPs were counted and measured.

### 2.4. Bacterial Strains Culture, DNA Extraction, and Double-Tagging PCR

*E. coli* DH5-α strain was cultured in Luria–Bertani (LB) plates for 16 h at 37 °C. After the overnight culturing, the concentration of bacteria was found to be approximately 10^8^ CFU·mL^−1^. Lysis of bacteria and DNA extraction were performed, incubating 1 mL of bacteria at 99 °C for 1 h at different concentrations and centrifuging at 15,500× *g* for 2 min. The lysate was washed with Milli-Q water, resuspended with 200 μL Tris-EDTA (10 mmol·L^−1^ Tris; 1 mmol·L^−1^; pH 8.0) for DNA extraction, and incubated for 10 min at 99 °C followed by 15 min in ice. Genomic DNA was centrifuged for 5 min at 12,000× *g* and resuspended at different concentrations.

### 2.5. Double-Tagging PCR and Quantification by Gel Electrophoresis

**Figure 1:**
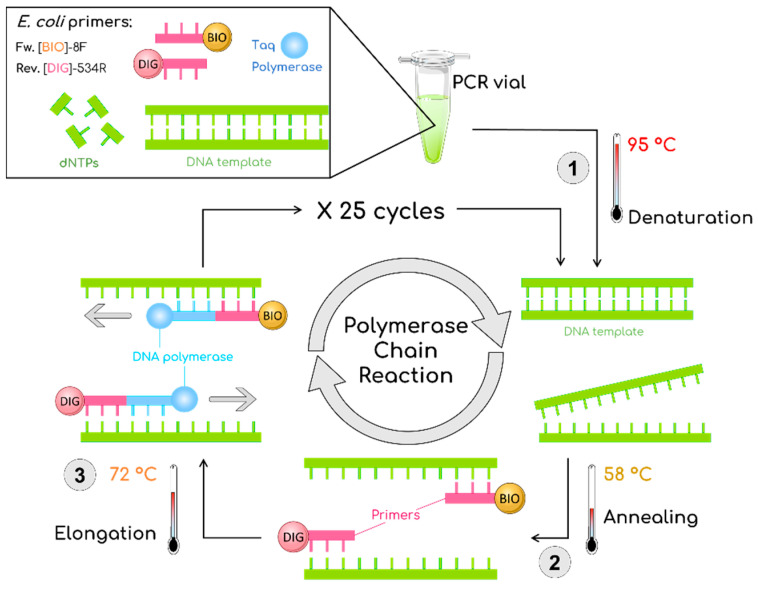
Schematic description of the double-tagging PCR for the amplification of the *Escherichia coli* 16S ribosomal gene, including an initial step at 95 °C for 3 min followed by 25 cycles of 95 °C for 30 s, 58 °C for 30 s, 72 °C for 30 s, and a final step of 7 min at 72 °C. BIO is biotin marked with orange; DIG is digoxigenin marked with pink.

During the double-tagging PCR procedure (Figure 1), bacterial DNA was amplified and labeled with biotin and digoxigenin using modified primers [29,30]. PCR was carried out in 15 μL reactions. The primers for the double-tagging PCR were selected for the specific amplification of the *E. coli* 16S ribosomal gene (NCBI Reference Sequence MN661169.1, as detailed in Supplementary Materials). The sequences of the primers were as shown in Table 1.

Further experimental details of the double-tagging PCR procedure are also described in the Supplementary Materials.

Afterward, amplimers were analyzed with conventional agarose gel electrophoresis on 2% agarose gel in TAE buffer (40 mmol·L^−1^ Tris, 40 mmol·L^−1^ acetate, 1 mmol·L^−1^ EDTA, containing a DNA stain to check its size distribution.

### 2.6. Lateral Flow Assays Based on Carbon and Gold Nanoparticles as Signal-Generating Systems

**Figure 2:**
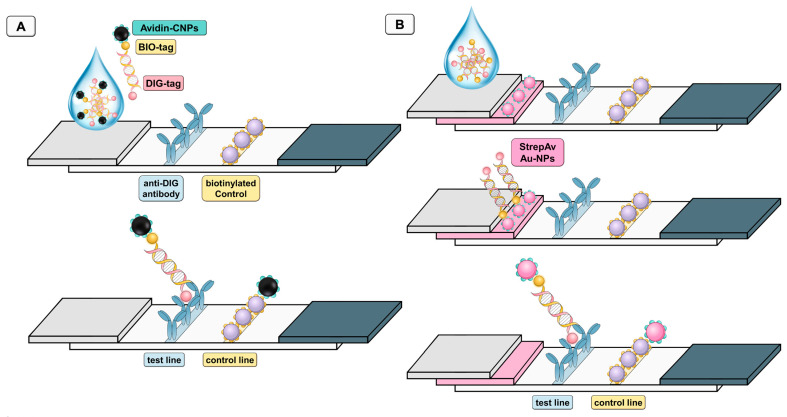
Schematic procedure of the lateral flow assay (LFA) based on avidin-modified carbon nanoparticles (Av-CNPs) (**A**) and based on commercial streptavidin gold nanoparticles, (streptAv-AuNPs) (**B**) for the detection of double-tagged amplicon labeled with biotin and digoxigenin. The test line is based in both instances on anti-digoxigenin antibody (anti-DIG antibody), while the control line is based on a biotinylated protein, so as to capture all the remaining Av-CNPs (**A**) or streptavidin-modified AuNPs (**B**).

Figure 2 shows the configuration of Av-CNPs (Figure 2A) and streptavidin-modified AuNPs (Figure 2B) lateral flow immunoassays. In both instances, the biotin tag of the double-tagged amplicon reacted with the signal-generating systems, while the immobilization of the DNA was achieved by the digoxigenin tag and using anti-DIG antibody on the nitrocellulose strip in the test line. As a control line, a biotinylated macromolecule (such as biotinylated protein or biotinylated dendrimer as previously reported by our research group) [31,32] can be used in order to capture all the remaining Av-CNPs, as shown in Figure 2. It is important to highlight that biotin alone cannot be used, since it is not adsorbed on nitrocellulose strips due to its low MW and, as such, it is washed during the test, as previously reported by our research group [32]. In this instance, a biotinylated protein (a biotin-modified rabbit polyclonal antibody) was used.

In the CNP strips, 5 μL of Av-CNPs was added to the sample in a final volume of 50 μL running buffer (100 mmol·L^−1^ borate buffer, pH 8.8, with 1% (*w*/*v*) BSA, 0.05% (*v*/*v*) Tween^®^ 20), and was dropped into the sample pad after 2 min of incubation. A further 200 μL of running buffer was dropped into the sample pad. The readout was achieved after 10 min from sample deposition. For the semiquantitative plots, the samples are the double-tagged amplicons at different concentrations (from 0.00 to 7.09 pg μL^−1^/1.5 × 10^4^ CFU mL^−1^).

Additionally, AuNP-based lateral flow strips were performed as previously described by our research group [31,32], and included a conjugate pad in the configuration as a reservoir of AuNPs. In this instance, a final readout was achieved at 15 min from sample deposition.

### 2.7. Data Interpretation and Analysis

In order to avoid any bias in the images due to luminosity, all the strips for a single experiment were photographed in a single image and the conditions were the same in all cases. The images were taken at the same time for all the strips in a single image, using a portable photographic studio based on LED lights of 1100 lm and a color temperature of 6000–6500 K. The images were taken at a distance of 23 cm with a smartphone. The rear camera was used, with a maximum resolution of 12 megapixels (4032 × 3024 pixels). Autofocus was enabled, and the flashlight was turned off during the data acquisition procedure. Then, the images were converted to an 8-bit grey-scale format using the command Image > Type > 8-bit. The test and control lines were outlined using the rectangular selection tool, and the area under each peak was then numerically integrated using the ImageJ gel analysis toolbox.

## 3. Results and Discussion

### 3.1. Characterization of CNPs by Transmission Electron Microscopy

**Figure 3:**
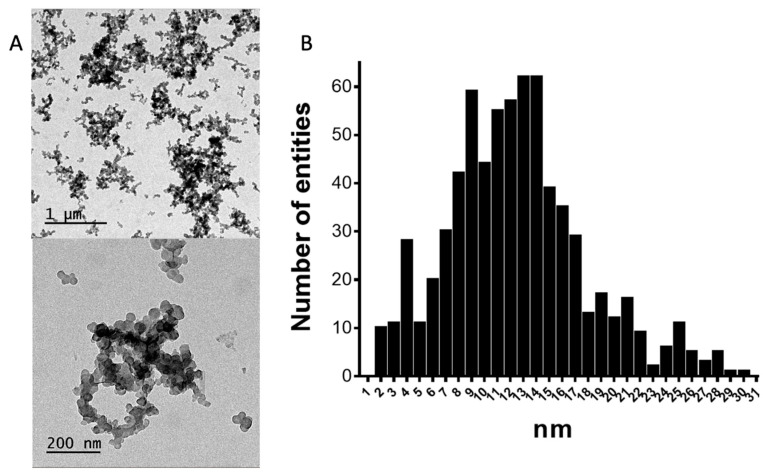
(**A**) The transmission electron microscopy (TEM) images obtained with a JEOL 1400 microscope at an accelerating voltage of 120 kV at two resolution levels. (**B**) The histogram obtained by analyzing 4 images (displayed in Figure S1, Supplementary Materials). *n* = 700.

The structure of the CNPs in CB is composed of a system of condensed carbon aromatic rings that are deposited forming sheets of different sizes and alignment. These sheets are placed randomly through an axis and are attached to each other by π−π stacking, overlapping one another to form the primary unit of the structure, the primary particle or nodule [13,14,15,16,17,18]. Besides the nodules, CNPs in CB tend to form aggregates (85–500 nm) and agglomerates (1–100 μm) [13,14,15,16,17,18]. After the sonication procedure necessary to disperse the CNPs in water, the remaining superstructures are primarily the aggregates, as shown in Figure 3A. The agglomerates are attached by weaker forces that can be broken by the sonication. The mean diameter value was found to be 13 nm with a standard deviation of 6 (Figure 3B). The TEM image shows the presence of aggregates without any regular structure that were, in most instances, larger than 200 nm.

### 3.2. Double-Tagging PCR and Quantification by Gel Electrophoresis

**Figure 4:**
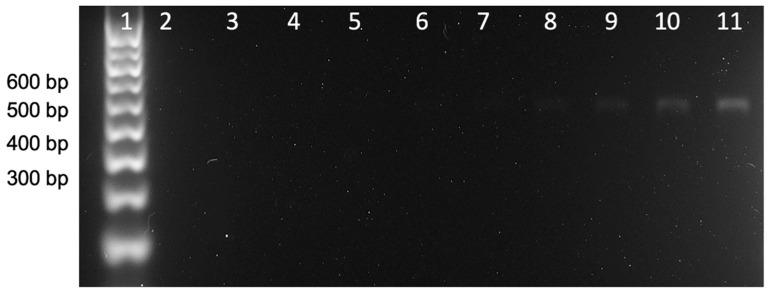
Gel electrophoresis of the double-tagging PCR amplification of the *E. coli* 16S ribosomal gene, including an initial step at 95 °C for 3 min followed by 25 cycles of 95 °C for 30 s, 58 °C for 30 s, 72 °C for 30 s, and a final step of 7 min at 72 °C. The amplification bands correspond to the *E. coli* 16S ribosomal gene (527 bp). Lane 1 is the molecular weight marker. Lane 2 corresponds to the negative control in which no DNA template has been added to the PCR mixture, while lanes 3–11 show the amplicon corresponding to DNA template concentrations at 0.1, 0.2, 0.4, 0.9, 1.8, 3.5, 7.1, 17.7, and 35.5 pg μL^−1^.

Figure 4 shows the results of the end-point double-tagging PCR analyzed by gel electrophoresis. As can be observed, a unique band at the expected MW (527) was obtained. Further detail of the *E. coli* 16S ribosomal RNA gene (partial sequence, region from 1 to 530 nt) is shown in Supplementary Materials, including the position of the forward and reverse primer sets framing the region to be amplified (527 bp). As shown in Figure 4, the visual limit of detection can be defined at line 8, corresponding to 3.5 pg μL^−1^.

### 3.3. Lateral Flow Assays Based on Carbon and Gold Nanoparticles as Signal-Generating Systems

**Figure 5:**
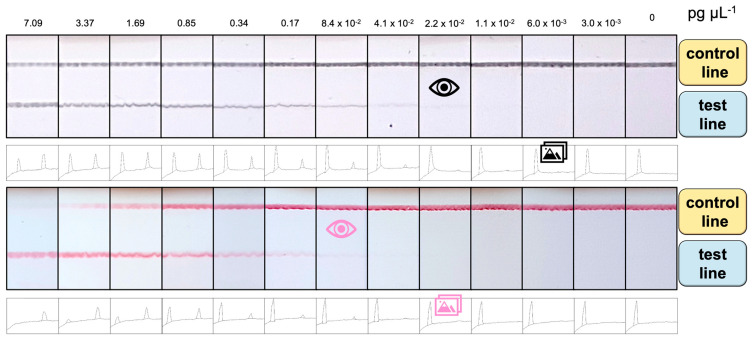
Results obtained for the LFA at template amounts ranging from 0.00 to 7.09 pg μL^−1^ (0 to 1.5 × 10^4^ CFU mL^−1^) of *E. coli*, based on Av-CNPs (upper panel) and streptAv-AuNPs (lower panel). The relative areas obtained after processing the images, plotted in Figure 6, are also shown for both signal-generating systems. The eye icon indicates the visual LODs, while the photo icon indicates the LOD obtained by processing the images and determining the areas.

**Figure 6:**
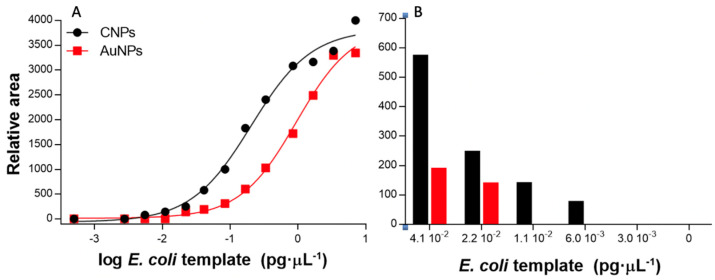
(**A**) The results obtained for the LFA at template concentrations ranging from 0.00 to 7.09 pg μL^−1^ (0 to 1.5 × 10^4^ CFU mL^−1^) of *E. coli*, based on carbon nanoparticles (CNPs) (•) and gold nanoparticles (AuNPs) (■). (**B**) Details of the relative areas at low concentration.

The comparative study for the detection of *E. coli* by double-tagging PCR followed by an LFA based on different signal-generating systems was performed, as schematically shown in Figure 2. The total assay time was less than 15 min. The results of the tests can either be estimated with the naked eye (as shown in Figure 5, the upper panel for Av-CNP and the lower panel for streptAv-AuNP) or with the ImageJ software by processing the images. Figure 5 also shows the relative areas for each type of signal-generating system after processing the images. These areas were used for Figure 6. The visual LOD of the method was calculated by serial dilution of the DNA template, ranging from 0.0 to 7 pg μL^−1^. The LFA achieved visual detection as low as 2.2 × 10^−2^ pg μL^−1^ using CNPs and 8.4 × 10^−2^ pg μL^−1^ using AuNPs. These LODs (calculated as the concentration corresponding to the last positive line visible to the naked eye, indicated by the eye icon in Figure 5) can be obtained without the assistance of any instrumentation but is prone to subjectivity.

To further analyze the strips and to avoid subjectivity, the ImageJ analysis tool was used to extract the line intensities from the images of the strips at different concentrations by using a smartphone. The area under the peak (both the intensities of the control and the test line for each system are shown in Figure 5) was then numerically integrated using the ImageJ analysis toolbox, and the intensity of the test line for each strip was fitted using non-linear regression (Sigmoidal dose–response variable slope/Sigmoidal 4 PL) (R^2^ = 0.992 and 0.995 for CNPs and AuNPs, respectively); the results are shown in Figure 6A.

The semiquantitative LODs were determined by processing the relative areas for each type of signal-generating system after processing the images, as shown in Figure 6. The LODs are calculated as the last concentration, in which a positive area value is obtained, as shown in Figure 5, indicated by a photo icon.

The LODs obtained showed improved values compared to the visual LOD. In the case of CNPs, the LOD was found to be 6.0 10^−3^ pg μL^−1^ (which corresponds to 10 CFU mL^−1^), much better than the visual LOD of 2.2 10^−2^ pg μL^−1^ (as shown in Figure 6B). Similarly, the semiquantitative LOD was 2.2 10^−2^ for the AuNPs, while the visual LOD was found to be 8.4 10^−2^ pg μL^−1^.

Besides the improved LODs obtained with the CNPs, the valid analysis range was also found to be wider (from 0 to at least 7.09 pg μL^−1^), since valid tests (with a positive control line) were obtained in all instances, at least up to this concentration. As shown in Figure 5, a clear control line was obtained with CNPs at this concentration. Whereas, at 7.09 pg μL^−1^, a non-valid test was observed by the AuNPs system since the control line was negative.

**Figure 7:**
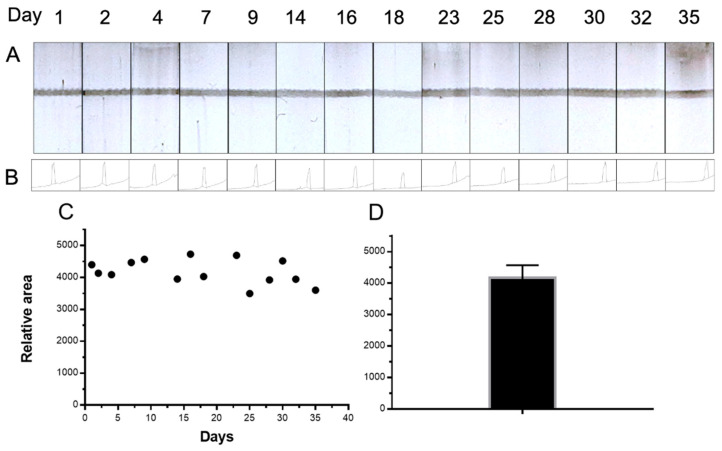
(**A**) The stability and repeatability study of the strips based on Av-CNP, performed with the control line for 35 days. The signal values for the relative areas are also shown in (**B**). (**C**) The plot of the signal intensities along with times of up to 35 days, while the mean value of the signal intensities’ areas is displayed in (**D**). The error bar shows the standard deviation for *n* = 14.

The stability and repeatability study of Av-CNPs is shown in Figure 7. This study was performed with the strips prepared on day 0 and stored at room temperature. Moreover, a batch of Av-CNPs was also prepared on day 0 and stored at 4 °C. This study was conducted for 35 days (Figure 7C). The results show a mean relative intensity value of 4179, a standard deviation of 389, and a relative standard deviation (RSD) of 9.3% (as shown in Figure 7D). This error shows the repeatability study, including the construction of the strips, the deposition of the line, and the stability of the Av-CNPs. As shown in Figure 7, the Av-CNPs were able to produce a clear control line until day 35, demonstrating outstanding stability at least a month after preparation.

## 4. Conclusions

In this paper, the conjugation of avidin to carbon black nanoparticles and the design of LFAs for the detection of PCR products are presented in order to compare the performance of Av-CNP to the well-known commercial gold nanoparticles in optimal conditions for both cases.

The results of the TEM characterization of the CNPs revealed the existence of aggregates and some primary particles following sonication. The sonication is necessary in order to dissolve the particles.

The conjugated CNPs showed good sensitivity and specificity for biotinylated amplicons, as confirmed also by the control line using an adsorbed biotinylated protein. Moreover, the conjugated CNPs showed non-detectable non-specific adsorption, since no signal was observed in the negative test, as in the case of the commercial streptAv-AuNP. The performance of Av-CNPs in this LFA format reveals promising features for use in other applications, enabling a rapid visual readout at an impressive LOD.

The comparative results of Av-CNPs and the commercial streptAv-AuNPs indicated improved LODs for Av-CNPs under the experimental conditions of this work, which can be attributed to the higher signal background contrasts, considering the intensity of the black color. All these features make the CNPs an attractive option to work with and to improve the sensitivity, cost, and simplicity of lateral flow tests.

## Figures and Tables

**Table 1:** Sequences of the set of primers for the double-tagging PCR amplification of *E. coli*.

Strain and Gene	Primer Sequence	Type	5′-Labels	Size (bp)
*Escherichia coli* DH5-α 16S ribosomal gene	AGAGTTTGATCCTGGCTCAG	Forward	biotin	527
	ATTACCGCGGCTGCTGGC	Reverse	digoxigenin	

## Data Availability

No new data were created or analyzed in this study. Data sharing is not applicable to this article.

## Supplementary Materials

The following are available online at https://www.mdpi.com/2079-4991/11/3/741/s1, Figure S1: The four transmission Electron Microscopy images used to perform the histogram shown in Figure 3.
