# Supplementary material for: Comparative Study of Gold and Carbon Nanoparticles in Nucleic Acid Lateral Flow Assay
Source: Nanomaterials (Basel). 2021 Mar 15;11(3):741. doi: 10.3390/nano11030741 (PMC8000918; doi:10.3390/nano11030741)
Supplement: Supplementary file 1 [file nanomaterials-11-00741-s001.pdf]

nanomaterials MDPI

Supplementary Materials

Comparative Study of Gold and Carbon Nanoparticles in Nucleic Acid Lateral Flow Assay

Juan Carlos Porras [1], Mireia Bernuz [1], Jennifer Marfa [1], Arnau Pallares-Rusiñol [1], Mercè Martí [2] and María Isabel Pividori [1,2,*]

[1] Grup de Sensors i Biosensors, Departament de Química, Universitat Autònoma de Barcelona, 08193 Bellaterra, Spain; juancarlos.porras@e-campus.uab.cat (J.C.P.); Mireia.Bernuz@uab.cat (M.B.); Jennifer.Marfa@uab.cat (J.M.); Arnau.Pallares@uab.cat (A.P.-R.)
[2] Institute of Biotechnology and Biomedicine, Universitat Autònoma de Barcelona, 08193 Bellaterra, Spain; merce.marti@uab.cat
* Correspondence: Isabel.Pividori@uab.cat; Tel.: +34-93-581-1976

2. Materials and Methods

2.1. Reagents and Equipment

2.2.1. Buffers and Solutions

Tris-EDTA: 10 mmol·L^{-1} TRIS, 1 mmol·L^{-1} EDTA, pH 8.0

For the preparation, storage and use of Avidin-modified Carbon Nanoparticles (CNPs), the buffers are:

- Borate buffer 1:100 mmol·L^{-1} borate, pH 8.8
- Borate buffer 2: 5 mmol·L^{-1} borate, pH 8.8.
- Washing buffer: 5 mmol·L^{-1} borate, pH 8.8, 1% (*w*/*v*) BSA.
- Storage buffer: 100 mmol·L^{-1} borate, pH 8.8, 1% (*w*/*v*) BSA.
- Running buffer: 100 mmol·L^{-1} borate, pH 8.8, 1% (*w*/*v*) BSA, 0.05% (*v*/*v*) Tween 20®.

For the use of Streptavidin-modified Gold nanoparticles (Au-NPs), the buffers were:

- Conjugate buffer: 10 mmol·L^{-1} borate, pH 8.8, 20% (*w*/*v*) sucrose.
- Running buffer: PBS 10 mmol·L^{-1}, pH 7.4, 0.05% (*v*/*v*) Tween 20®, 1% (*w*/*v*) BSA.

All solutions were prepared with ultrapure MilliQ water (Millipore® System, resistivity 18.2 MΩ·cm).

2.5. Double-Tagging PCR and Quantification by Gel Electrophoresis

2.5.1. DNA Amplimer Sequence

The forward sequence is highlighted in orange, while the reverse sequence is in pink.
NCBI Reference Sequence: MN661169.1

Escherichia coli strain FC5906 16S ribosomal RNA gene, partial sequence. Region from 1 to 530 nt.

```
AGAGTTTGAT CCTGGCTCAG ATTGAACGCT GGCGGCAGGC CTAACACATG CAAGTCGAAC GGTAACAGGA AGAAGCTTGC
TTCTTTGCTG ACGAGTGGCG GACGGGTGAG TAATGTCTGG GAAACTGCCT GATGGAGGGG GATAACTACT GGAAACGGTA
GCTAATACCG CATAACGTCG CAAGACCAAA GAGGGGGACC TTCGGGCCTC TTGCCATCGG ATGTGCCCAG ATGGGATTAG
CTAGTAGGTG GGGTAACGGC TCACCTAGGC GACGATCCCT AGCTGGTCTG AGAGGATGAC CAGCCACACT GGAACTGAGA
CACGGTCCAG ACTCCTACGG GAGGCAGCAG TGGGGAATAT TGCACAATGG GCGCAAGCCT GATGCAGCCA TGCCGCGTGT
ATGAAGAAGG CCTTCGGGTT GTAAAGTACT TTCAGCGGGG AGGAAGGGAG TAAAGTTAAT ACCTTTGCTC ATTGACGTTA
CCCGCAGAAG AAGCACCGGC TAACTCCGTG CCAGCAGCCG CGGTAATACG
```

2.5.2. Procedure for the Double-Tagging PCR of E.Coli DNA

The double-tagging polymerase chain reaction (PCR) was performed in 15 µL of reaction mixture containing the DNA as sample in order to obtain the 16S ribosomal gene amplimers doubly labelled with biotin and digoxigenin. Each reaction mixture contained 7.5 pmol of each primer (BIO-8F and DIG-534R), 3.75 nmol of each deoxynucleotide triphosphate (dNTPs) and 3U of Taq polymerase. The reaction was carried out in a buffer with 7.5 mmol L^{-1} TRIS (pH 9.0), 5.0 mmol L^{-1} KCl, 2.0 mmol L^{-1} $(NH_4)_2SO_4$ and 0.2 mmol L^{-1} $MgCl_2$ as a cofactor of the enzyme. The reaction mixture was exposed to an initial step at 95 °C for 3 min followed by 25 cycles of 95 °C for 30 s, 58 °C for 30 s, 72 °C for 30 s, and a last step of 7 min at 72 °C.

Afterwards amplimers were analyzed with conventional agarose gel electrophoresis on 2% agarose (Sigma Aldrich, St. Louis, MO, USA, Ref. A9539) gel in TAE buffer (Fisher Scientific, Waltham, MA, USA, Ref. 1335) containing 1× SYBR Gold dye, loading buffer (Invitrogen, Ref. 10816015) and 100bp DNA ladder (Sigma Aldrich, St. Louis, MO, USA, Ref. D3687).

3. Results and Discussion

3.1. TEM Characterization

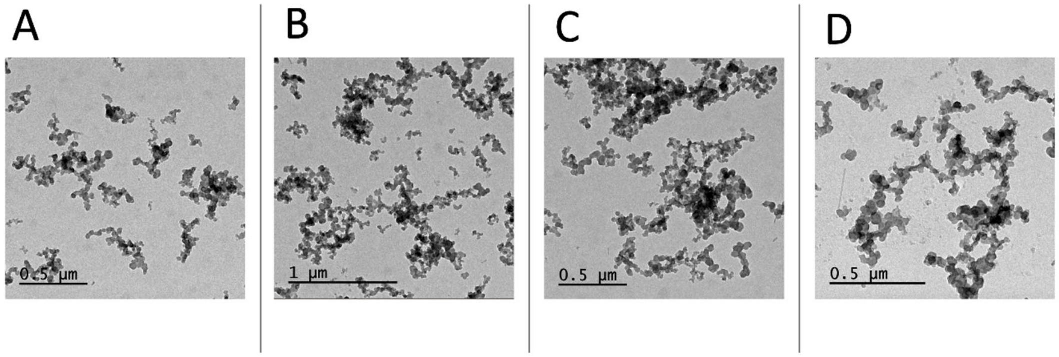

Figure S1. The four transmission Electron Microscopy images used to perform the histogram shown in Figure 3.
